# Genome sequence of *Ensifer* sp. TW10; a *Tephrosia wallichii* (Biyani) microsymbiont native to the Indian Thar Desert

**DOI:** 10.4056/sigs.4598281

**Published:** 2013-12-15

**Authors:** Nisha Tak, Hukam S Gehlot, Muskan Kaushik, Sunil Choudhary, Ravi Tiwari, Rui Tian, Yvette Hill, Lambert Bräu, Lynne Goodwin, James Han, Konstantinos Liolios, Marcel Huntemann, Krishna Palaniappan, Amrita Pati, Konstantinos Mavromatis, Natalia Ivanova, Victor Markowitz, Tanja Woyke, Nikos Kyrpides, Wayne Reeve

**Affiliations:** 1 BNF and Stress Biology Lab, Department of Botany, JNV University, Jodhpur, India; 2 Centre for Rhizobium Studies, Murdoch University, Western Australia, Australia; 3 School of Life and Environmental Sciences, Faculty of Science, Engineering and Built Environment, Deakin University, Melbourne, Victoria, Australia; 4 Los Alamos National Laboratory, Bioscience Division, Los Alamos, New Mexico, USA; 5 DOE Joint Genome Institute, Walnut Creek, California, USA; 6 Biological Data Management and Technology Center, Lawrence Berkeley National Laboratory, Berkeley, California, USA

**Keywords:** root-nodule bacteria, nitrogen fixation, rhizobia, *Alphaproteobacteria*

## Abstract

*Ensifer* sp. TW10 is a novel N_2_-fixing bacterium isolated from a root nodule of the perennial legume *Tephrosia wallichii* Graham (known locally as Biyani) found in the Great Indian (or Thar) desert, a large arid region in the northwestern part of the Indian subcontinent. Strain TW10 is a Gram-negative, rod shaped, aerobic, motile, non-spore forming, species of root nodule bacteria (RNB) that promiscuously nodulates legumes in Thar Desert alkaline soil. It is fast growing, acid-producing, and tolerates up to 2% NaCl and capable of growth at 40^o^C. In this report we describe for the first time the primary features of this Thar Desert soil saprophyte together with genome sequence information and annotation. The 6,802,256 bp genome has a GC content of 62% and is arranged into 57 scaffolds containing 6,470 protein-coding genes, 73 RNA genes and a single rRNA operon. This genome is one of 100 RNB genomes sequenced as part of the DOE Joint Genome Institute 2010 Genomic Encyclopedia for *Bacteria* and *Archaea*-Root Nodule Bacteria (GEBA-RNB) project.

## Introduction

The Great Indian (or Thar) Desert is a large, hot, arid region in the northwestern part of the Indian subcontinent. It is the 18th largest desert in the world covering 200,000 square km with 61% of its landmass occupying Western Rajasthan. The landscape occurs at low altitude (<1500 m above sea level) and extends from India into the neighboring country of Pakistan [1]. The Thar Desert region is characterized by low annual precipitation (50 to 300 mm), high thermal load and alkaline soils that are poor in texture and fertility [2]. Despite these harsh conditions, the Thar Desert has very rich plant diversity in comparison to other desert landscapes [3]. Approximately a quarter of the plants in the Thar Desert are used to provide animal fodder or food, fuel, medicine or shelter for local inhabitants [4].

The Indian Thar desert harbors several native and exotic plants of the *Leguminoseae* family [2] including native legume members of the sub-families *Caesalpinioideae*, *Mimosoideae* and *Papilionoideae* that have adapted to the harsh Thar desert environment [5]. The Papilionoid genus *Tephrosia* can be found throughout this semi-arid to arid environment and these plants are among the first to grow after monsoonal rains. The generic name is derived from the Greek word “tephros” meaning “ash-gray” since dense trichomes on the leaves provide a greyish tint to the plant. Many species within this genus produce the potent toxin rotenone, which historically has been used to poison fish. It is a perennial shrub that has adapted to the harsh desert conditions by producing a long tap root system and dormant auxillary shoot buds.

Recently, the root nodule bacteria (RNB) microsymbionts capable of fixing nitrogen in symbiotic associations with *Tephrosia* have been characterized [5]. Both *Bradyrhizobium* and *Ensifer* were present within nodules, but a particularly high incidence of *Ensifer* was noted [5]. *Ensifer* was found to occupy the nodules of all four species of *Tephrosia* examined [5]. Here we present a preliminary description of the general features of the *T*. *wallichii* (Biyani) microsymbiont *Ensifer* sp. TW10 together with its genome sequence and annotation.

**Figure 1:**
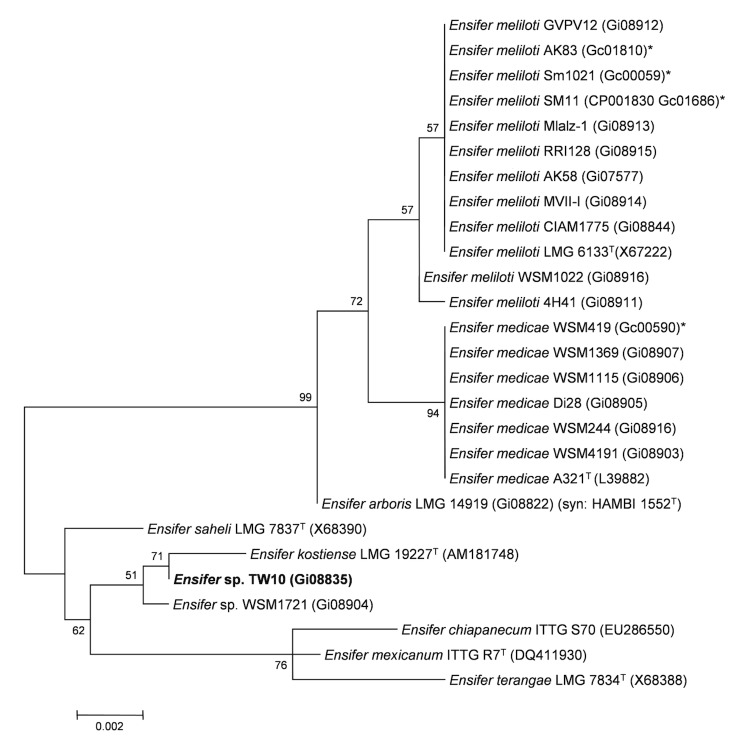
Phylogenetic tree showing the relationship of *Ensifer* sp. TW10 (shown in bold print) to other *Ensifer* spp. in the order *Rhizobiales* based on aligned sequences of the 16S rRNA gene (1,290 bp internal region). All sites were informative and there were no gap-containing sites. Phylogenetic analyses were performed using MEGA, version 5 [19]. The tree was built using the Maximum-Likelihood method with the General Time Reversible model [20]. Bootstrap analysis [21] with 500 replicates was performed to assess the support of the clusters. Type strains are indicated with a superscript T. Brackets after the strain name contain a DNA database accession number and/or a GOLD ID (beginning with the prefix G) for a sequencing project registered in GOLD [22]. Published genomes are indicated with an asterisk.

Minimum Information about the Genome Sequence (MIGS) is provided in Table 1. Figure 1 shows the phylogenetic neighborhood of *Ensifer* sp. strain TW10 in a 16S rRNA sequence based tree. This strain has 99% sequence identity at the 16S rRNA sequence level to *E*. *kostiense* LMG 19227 and 100% 16S rRNA sequence identity to other Indian Thar Desert *Ensifer* species (JNVU IC18 from a nodule of *Indigofera* and JNVU TF7, JNVU TP6 and TW8 from nodules of *Tephrosia*).

**Table 1:** Classification and general features of *Ensifer* sp. TW10 according to the MIGS recommendations [6]

**MIGS ID**	**Property**	**Term**	**Evidence code**
	Current classification	Domain *Bacteria*	TAS [7]
		Phylum *Proteobacteria*	TAS [8]
		Class *Alphaproteobacteria*	TAS [9,10]
		Order *Rhizobiales*	TAS [10,11]
		Family *Rhizobiaceae*	TAS [12,13]
		Genus *Ensifer*	TAS [14-16]
		Species *Ensifer sp.*	IDA
	
	Gram stain	Negative	IDA
	Cell shape	Rod	IDA
	Motility	Motile	IDA
	Sporulation	Non-sporulating	NAS
	Temperature range	Mesophile	NAS
	Optimum temperature	28°C	NAS
	Salinity	Non-halophile	NAS
MIGS-22	Oxygen requirement	Aerobic	TAS [5]
	Carbon source	Varied	NAS
	Energy source	Chemoorganotroph	NAS
MIGS-6	Habitat	Soil, root nodule, on host	TAS [5]
MIGS-15	Biotic relationship	Free living, symbiotic	TAS [5]
MIGS-14	Pathogenicity	Non-pathogenic	NAS
	Biosafety level	1	TAS [17]
	Isolation	Root nodule of *Tephrosia wallichii*	TAS [5]
MIGS-4	Geographic location	Jodhpur, Indian Thar Desert	TAS [5]
MIGS-5	Soil collection date	Oct, 2009	IDA
MIGS-4.1	Longitude	73.021177	IDA
MIGS-4.2	Latitude	26.27061	IDA
MIGS-4.3	Depth	15cm	
MIGS-4.4	Altitude	Not recorded	

Evidence codes – IDA: Inferred from Direct Assay; TAS: Traceable Author Statement (i.e., a direct report exists in the literature); NAS: Non-traceable Author Statement (i.e., not directly observed for the living, isolated sample, but based on a generally accepted property for the species, or anecdotal evidence). These evidence codes are from the Gene Ontology project [18].

## Classification and general features

**Figure 2:**
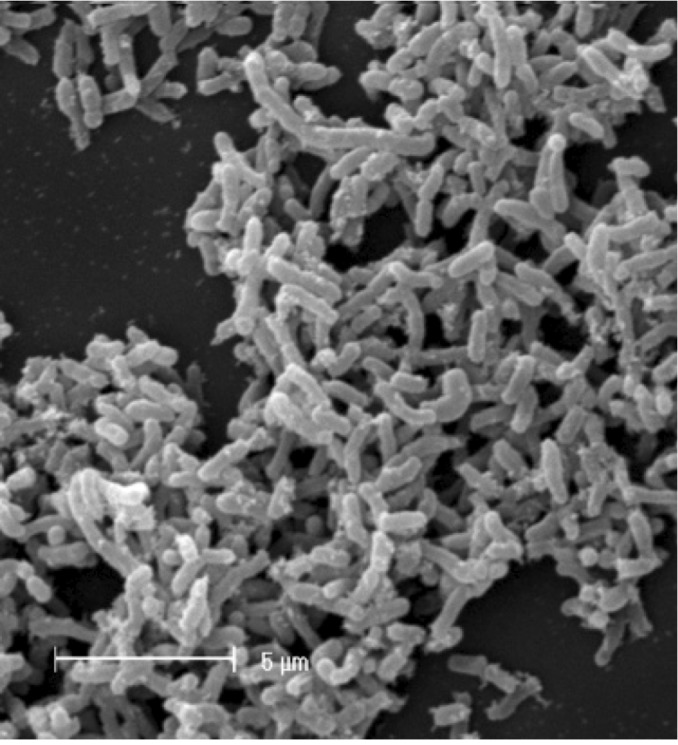
Image of *Ensifer* sp. TW10 using scanning electron microscopy.

**Figure 3:**
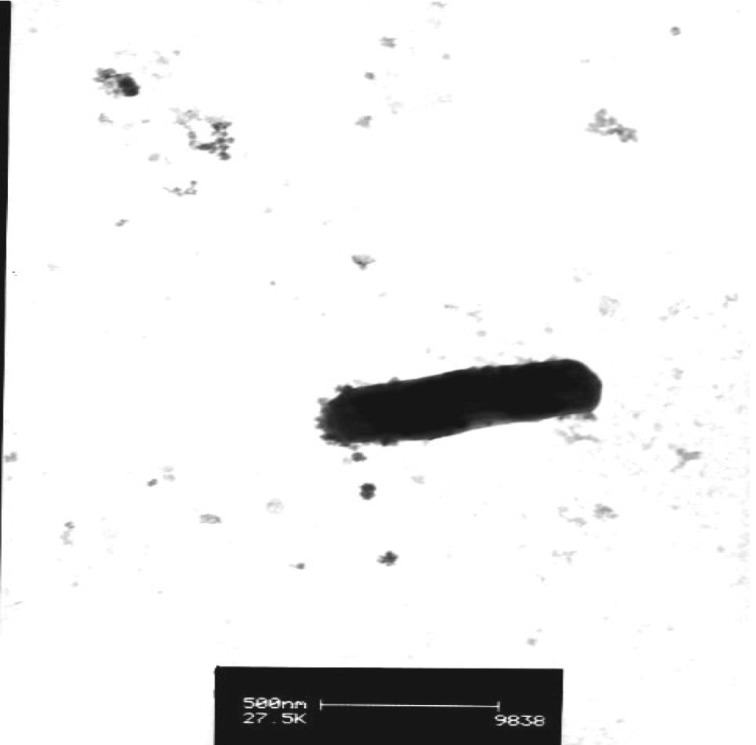
Image of *Ensifer* sp. TW10 using transmission electron microscopy.

*Ensifer* sp. strain TW10 is a Gram-negative rod (Figure 2, and Figure 3) in the order *Rhizobiales* of the class *Alphaproteobacteria*. It is fast growing, forming white-opaque, slightly domed and moderately mucoid colonies with smooth margins within 3-4 days at 28°C when grown on YMA [23].

### Symbiotaxonomy

*Ensifer* sp. TW10 has the ability to nodulate (Nod^+^) and fix nitrogen (Fix^+^) effectively with a wide range of perennial native (wild) legumes of Thar Desert origin and with species of crop legumes (Table 2). *Ensifer* sp. TW10 is symbiotically competent with these species when grown in alkaline soils. TW10 can nodulate the wild tree legume *Prosopis cineraria* of the *Mimosoideae* subfamily. However, it does not form nodules on the Mimosoid hosts *Mimosa hamata* and *M. himalayana* even though these hosts are known to be nodulated by *Ensifer* species [5,24]. TW10 was not compatible with the host *Phaseolus vulgaris*, a legume of the *Phaseolae* tribe.

**Table 2:** Compatibility of *Ensifer* sp. TW10 with different wild and cultivated legume species

**Species Name**	**Family**	**Wild/ Cultivar**	**Common Name**	**Habit/ Growth Type**	**Nod**	**Fix**
*Tephrosia falciformis* Ramaswami	*Papilionoideae*	Wild	Rati biyani	Under-shrub Perennial	+	+
*Tephrosia purpurea* (L.) Pers. sub sp. *leptostachya* DC.	*Papilionoideae*	Wild	-	Herb Annual/ Perennial	+	+
*Tephrosia purpurea* (L.) Pers. sub sp. *purpurea* (L.) Pers	*Papilionoideae*	Wild	Biyani, Sarphanko	Herb Annual/ Perennial	+	+
*Tephrosia villosa* (Linn.) Pres.	*Papilionoideae*	Wild	Ruvali-biyani	Herb Annual/ Perennial	+	+
*Prosopis cineraria* (Linn.) Druce.	*Mimosoideae*	Wild/ Cultivar	Khejari	Tree Perennial	+	+
*Mimosa hamata* Willd.	*Mimosoideae*	Wild	Jinjani, Jinjanio	Shrub Perennial	-	-
*M. himalayana* Gamble	*Mimosoideae*	Wild	Hajeru	Shrub Perennial	-	-
*Vigna radiata* (L.) Wilczek	*Papilionoideae*	Cultivar	Moong bean	Annual	+	+
*Vigna aconitifolia* (Jacq.) Marechal	*Papilionoideae*	Cultivar	Moth bean	Annual	+	+
*Vigna unguiculata* (L.) Walp.	*Papilionoideae*	Cultivar	Cowpea	Annual	+	+
*Macroptilium atropurpureum* (DC.) Urb.	*Papilionoideae*	Cultivar	Siratro	Annual	+	+
*Phaseolus vulgaris* L.	*Papilionoideae*	Cultivar	Common bean	Annual	-	-

Nod: “+” means nodulation observed, “-” means no nodulation

Fix: “+” means fixation observed, “-” means no fixation

## Genome sequencing and annotation

### Genome project history

This organism was selected for sequencing on the basis of its environmental and agricultural relevance to issues in global carbon cycling, alternative energy production, and biogeochemical importance, and is part of the Community Sequencing Program at the U.S. Department of Energy, Joint Genome Institute (JGI) for projects of relevance to agency missions. The genome project is deposited in the Genomes OnLine Database [22] and standard draft genome sequence in IMG. Sequencing, finishing and annotation were performed by the JGI. A summary of the project information is shown in Table 3.

**Table 3:** Genome sequencing project information for *Ensifer* sp. strain TW10.

**MIGS ID**	**Property**	**Term**
MIGS-31	Finishing quality	Standard draft
MIGS-28	Libraries used	1× Illumina library
MIGS-29	Sequencing platforms	Illumina HiSeq2000
MIGS-31.2	Sequencing coverage	330× Illumina
MIGS-30	Assemblers	Allpaths, LG version r42328, Velvet 1.1.04
MIGS-32	Gene calling methods	Prodigal 1.4,
	GenBank Genbank Date of Release GOLD ID	pending pending Gi08835
	NCBI project ID	210334
	Database: IMG	2509276019
	Project relevance	Symbiotic N_2_ fixation, agriculture

### Growth conditions and DNA isolation

*Ensifer* sp. TW10 was cultured to mid logarithmic phase in 60 ml of TY rich medium [25] on a gyratory shaker at 28°C. DNA was isolated from the cells using a CTAB (Cetyl trimethyl ammonium bromide) bacterial genomic DNA isolation method [26].

### Genome sequencing and assembly

The genome of *Ensifer* sp. TW10 was generated at the Joint Genome Institute (JGI) using Illumina [27] technology. An Illumina std shotgun library was constructed and sequenced using the Illumina HiSeq 2000 platform which generated 14,938,244 reads totaling 2,241 Mbp.

All general aspects of library construction and sequencing performed at the JGI can be found at the JGI website [26]. All raw Illumina sequence data was passed through DUK, a filtering program developed at JGI, which removes known Illumina sequencing and library preparation artifacts (Mingkun L, Copeland, A, and Han, J, unpublished).

The following steps were then performed for assembly: (1) filtered Illumina reads were assembled using Velvet [28] (version 1.1.04), (2) 1–3 kb simulated paired end reads were created from Velvet contigs using wgsim (https://github.com/lh3/wgsim), and (3) Illumina reads were assembled with simulated read pairs using Allpaths–LG (version r42328) [29]. Parameters for assembly steps were: 1) Velvet (velveth: 63 –shortPaired and velvetg: –veryclean yes –exportFiltered yes –mincontiglgth 500 –scaffolding no–covcutoff 10) 2) wgsim (–e 0 –1 100 –2 100 –r 0 –R 0 –X 0) 3) Allpaths–LG (PrepareAllpathsInputs:PHRED64=1 PLOIDY=1 FRAGCOVERAGE=125 JUMPCOVERAGE=25 LONGJUMPCOV=50, RunAllpath-sLG: THREADS=8 RUN=stdshredpairs TARGETS=standard VAPIWARNONLY=True OVERWRITE=True). The final draft assembly contained 57 contigs in 57 scaffolds. The total size of the genome is 6.8 Mbp and the final assembly is based on 2241Mbp of Illumina data, which provides an average 330× coverage of the genome.

### Genome annotation

Genes were identified using Prodigal [30] as part of the DOE-JGI annotation pipeline [31]. The predicted CDSs were translated and used to search the National Center for Biotechnology Information (NCBI) non-redundant database, UniProt, TIGRFam, Pfam, PRIAM, KEGG, COG, and InterPro databases. The tRNAScanSE tool [7] was used to find tRNA genes, whereas ribosomal RNA genes were found by searches against models of the ribosomal RNA genes built from SILVA [32]. Other non–coding RNAs such as the RNA components of the protein secretion complex and the RNase P were identified by searching the genome for the corresponding Rfam profiles using INFERNAL [33]. Additional gene prediction analysis and manual functional annotation was performed within the Integrated Microbial Genomes (IMG) platform) [34,35].

## Genome properties

**Figure 4:**
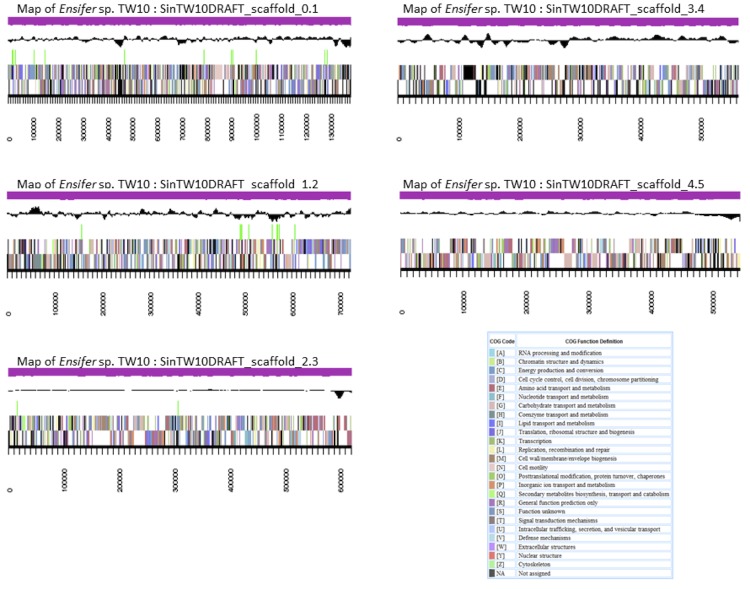
Graphical map of five of the largest scaffolds from the genome of *Ensifer* sp. TW10. From bottom to the top of each scaffold: Genes on forward strand (color by COG categories), Genes on reverse strand (color by COG categories), RNA genes (tRNAs green, sRNAs red, other RNAs black), GC content, GC skew.

The genome is 6,802,256 nucleotides with 61.56% GC content (Table 4) and comprised of 57 scaffolds (Figure 4) of 57 contigs. From a total of 6,546 genes, 6,473 were protein encoding and 73 RNA only encoding genes. The majority of genes (77.44%) were assigned a putative function while the remaining genes were annotated as hypothetical. The distribution of genes into COGs functional categories is presented in Table 5.

**Table 4:** Genome Statistics for *Ensifer* sp. TW10

**Attribute**	**Value**	**% of Total**
Genome size (bp)	6,802,256	100.00
DNA coding region (bp)	5,800,968	85.28
DNA G+C content (bp)	4,187,461	61.56
Number of scaffolds	57	
Number of contigs	57	
Total gene	6,546	100.00
RNA genes	73	1.12
rRNA operons	1	
Protein-coding genes	6,473	98.88
Genes with function prediction	5,069	77.44
Genes assigned to COGs	5,069	77.44
Genes assigned Pfam domains	5,282	80.69
Genes with signal peptides	539	8.23
Genes with transmembrane helices	1,419	21.68

**Table 5:** Number of protein coding genes of *Ensifer* sp. TW10 associated with the general COG functional categories.

Code	Value	%age	Description
J	198	3.55	Translation, ribosomal structure and biogenesis
A	0	0.00	RNA processing and modification
K	481	8.61	Transcription
L	237	4.24	Replication, recombination and repair
B	3	0.05	Chromatin structure and dynamics
D	37	0.66	Cell cycle control, mitosis and meiosis
Y	0	0.00	Nuclear structure
V	66	1.18	Defense mechanisms
T	262	4.69	Signal transduction mechanisms
M	298	5.34	Cell wall/membrane biogenesis
N	77	1.38	Cell motility
Z	0	0.00	Cytoskeleton
W	1	0.02	Extracellular structures
U	132	2.36	Intracellular trafficking and secretion
O	192	3.44	Posttranslational modification, protein turnover, chaperones
C	322	5.77	Energy production conversion
G	538	9.63	Carbohydrate transport and metabolism
E	606	10.85	Amino acid transport metabolism
F	96	1.72	Nucleotide transport and metabolism
H	194	3.47	Coenzyme transport and metabolism
I	199	3.56	Lipid transport and metabolism
P	251	4.49	Inorganic ion transport and metabolism
Q	139	2.49	Secondary metabolite biosynthesis, transport and catabolism
R	678	12.14	General function prediction only
S	578	10.35	Function unknown
-	1,477	22.56	Not in COGS
